# Association between the ABO blood group and the human intestinal microbiota composition

**DOI:** 10.1186/1471-2180-12-94

**Published:** 2012-06-06

**Authors:** Harri Mäkivuokko, Sampo J Lahtinen, Pirjo Wacklin, Elina Tuovinen, Heli Tenkanen, Janne Nikkilä, Marika Björklund, Kari Aranko, Arthur C Ouwehand, Jaana Mättö

**Affiliations:** 1 The Finnish Red Cross Blood Service, Helsinki, 00310, Finland; 2 Danisco Health & Nutrition, Kantvik, 02460, Finland

## Abstract

**Background:**

The mucus layer covering the human intestinal epithelium forms a dynamic surface for host-microbial interactions. In addition to the environmental factors affecting the intestinal equilibrium, such as diet, it is well established that the microbiota composition is individually driven, but the host factors determining the composition have remained unresolved.

**Results:**

In this study, we show that ABO blood group is involved in differences in relative proportion and overall profiles of intestinal microbiota. Specifically, the microbiota from the individuals harbouring the B antigen (secretor B and AB) differed from the non-B antigen groups and also showed higher diversity of the Eubacterium rectale-Clostridium coccoides (EREC) and Clostridium leptum (CLEPT) -groups in comparison with other blood groups.

**Conclusions:**

Our novel finding indicates that the ABO blood group is one of the genetically determined host factors modulating the composition of the human intestinal microbiota, thus enabling new applications in the field of personalized nutrition and medicine.

## Background

The human gastrointestinal tract (GIT) comprises an extremely dense and diverse microbiota. The GIT of an adult may harbour even 2 kg of bacterial biomass representing over 1000 bacterial species, of which majority can not be cultivated [1]. This microbiota in the large intestine is mainly composed of Firmicutes and Bacteroidetes phyla making up respectively over 75% and 16% of total microbes in the GIT [1]. The human intestinal microbiota has recently been shown to cluster into three distinct enterotypes [2] and of these enterotypes, *Bacteroides* and *Prevotella* dominated microbial communities have been reported to be associated with long-term diets [3]. Previously, twin studies have suggested a role for the host genotype in determining the microbiota composition [4], but the genetic host factors potentially affecting the gastrointestinal microbiota composition are unknown to a large extent.

The mucosal layer covering our gut epithelium has an important role as the first layer of host defences, but it also enables contacts between intestinal microbiota and the host [1,5]. The microbial biomass in the large intestine is mainly residing in the lumen and the mucosa-associated population differs from the lumen population [1]. There is a continuous interplay between the mucus secretion and degradation by bacteria as bacterial metabolites have been shown to act as signalling molecules modulating the mucus synthesis [6]. The mucus is mainly composed of mucins, large glycoproteins containing a protein core and attached oligosaccharides [7]. We recently observed a significant association between the blood group secretor status (encoded by fucosyltransferase-2, *FUT2,* gene) and the intestinal bifidobacteria composition [8]. The secretor status defines the expression of the ABO blood group antigens in the mucus of secretor individuals (80% of Western population). These antigens are expressed in the intestinal mucosal layer, and act as binding sites or carbon sources for the intestinal microbes, thereby providing a host-specific genetic agent affecting the microbiota composition [9,10].

Some microbes e.g. *Helicobacter pylori* and some other pathogenic bacteria and viruses have been shown to use ABO blood group antigens as adhesion receptors [11]. ABO antigen binding ability has reported also for *Lactobacillus* spp., which tend to adhere in a strain-specific manner [12]. Besides adhesion sites, secreted mucus provides endogenous substrate for bacteria. The mucus may be a major nutrient source in situations, where carbohydrates originating elsewhere are limited [13]. Some microbes e.g. bifidobacteria and *Bacteroides thetaiotaomicron* are also able to specifically utilize blood group antigens, e.g. the glycan structures of ABO antigens [14,15].

In the present study, we aimed to evaluate, whether there is a correlation between ABO blood group phenotype and relative proportions of the most abundant groups of healthy human gastrointestinal microbiota. We used several well characterised molecular and biochemical methods in order to address the hypothesis in deep detail. To our knowledge, this is the first study comparing the effects of human blood group phenotype with the intestinal microbiota composition.

## Results & discussion

**Figure 1:**
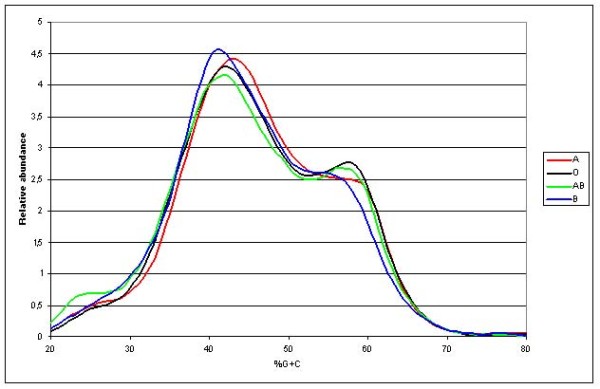
**%G + C-profile-data grouped by ABO blood groups.** Averaged %G + C-profiles grouped by ABO blood groups revealing a difference in the overall microbial profile between ABO blood groups. Each line represents the average of %G + C-data points of individuals with different ABO blood groups. Line colours for each ABO group are as follows: A = red, B = blue, AB = green and O = black.

In this study, we hypothesized that the ABO blood group antigens, which are expressed on the intestinal mucosa of secretor individuals [16,17] determine the gastrointestinal microbiota composition in healthy individuals. We recruited 79 healthy adult volunteers living in Southern Finland to test this hypothesis. The pool of study subjects was narrowed by excluding individuals with non-secretor phenotype and the fecal and blood samples of the final study pool of 64 volunteers was analysed by applying several molecular techniques (demographics in Figure 1). The male/female ratio in the final study pool was 7/57 (similar in each of the ABO blood groups), but as we were interested in the differences between the different ABO blood group antigens common to both genders and GIT microbiota, the gender imbalance was not considered to affect the results. Due to small number of subjects in each ABO blood group, no statistical methods were used to define the number of individuals in each of the study groups.

**Table 1:** Demographics of the study population

	**Blood group**			
	**A**	**B**	**AB**	**O**
Female	17 (85%)	11 (92%)	12 (92%)	17 (89%)
Male	3 (15%)	1 (8%)	1 (8%)	2 (11%)
Total*	20	12	13	19
Rh+	19 (95%)	10 (83%)	12 (92%)	19 (100%)
Rh-	1 (5%)	2 (17%)	1 (8%)	0
Average age**	44 (33–58)	43 (31–57)	48 (39–58)	46 (31–61)

79 persons were recruited to the study. Exclusion criteria in the recruitment were: diagnosed gastrointestinal disorders, antibiotic treatment in past two months, pregnancy, problems in blood coagulation, vegetarian diet and age below 18 or over 61. In addition, non-secretor persons (15) were excluded, thus the final study pool was 64 persons. Average age is presented together with the age range of each ABO blood group. Rh +/− states the presence/absence of the Rhesus-factor in blood.

*No statistical difference (P > 0.95) was detected in participant numbers between blood groups.

** No statistical difference (P > 0.45) was detected in participant age distribution between blood groups.

**Figure 2:**
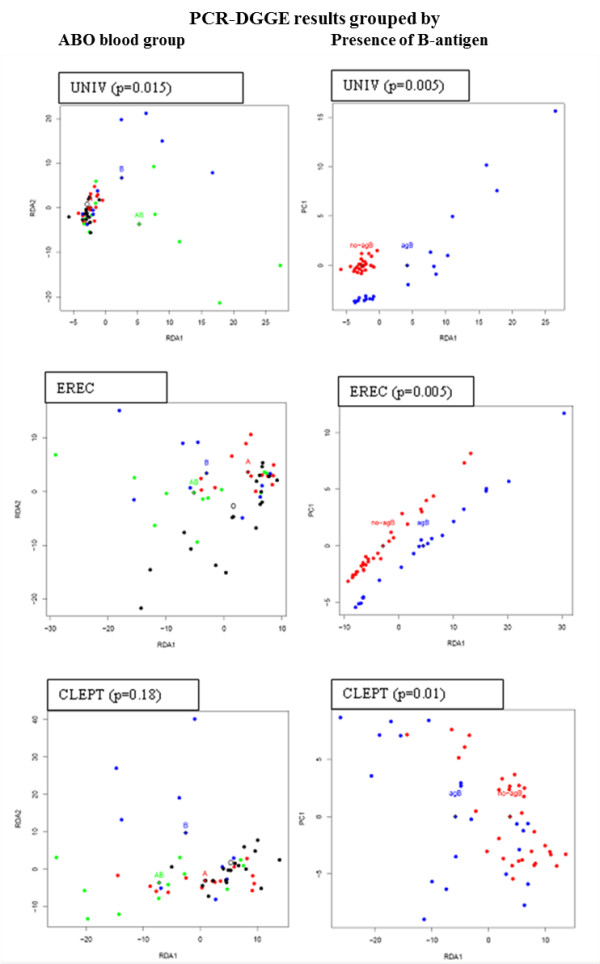
**RDA-visualization of PCR-DGGE profile similarities.** RDA visualization of microbiota profile similarities and ABO blood group types, revealing a clustering of the samples. Each dot represents a single individual and diamonds mark the calculated data centre points of the corresponding blood groups. *P*-value marks the statistical significance of the difference between blood group centres, computed with ANOVA-like permutation test from PCR-DGGE intensities grouped by ABO blood group (A) or by the presence of B-antigen (B). Dot colours for the ABO blood groups are as follows: A = red, B = blue, AB = green and O = black and for the B-antigen = blue and non-B antigen red, respectively. UNIV represent the PCR-DGGE obtained with the universal eubacterial primers (dominant bacteria), EREC with the *Eubacterium rectale* – *Clostridium coccoides* primers and CLEPT with the *Clostridium leptum* primers. The RDA analysis shows clustering of samples according to ABO blood groups, especially according to the presence of the B antigen in the dominant and EREC group microbiota.

The %G + C profiling that was performed to 46 fecal samples high enough genomic-DNA yield (>20 μg), revealed ABO blood group related differences in the overall faecal microbiota profiles (Figure1). The longitudinal shifts in the profile peaks suggested large differences in the microbiota composition, particularly evident in the mid-%G + C area (35–45; representing the majority of faecal microbes) and the high %G + C area (55–59; the area dominated by *Actinobacteria*). In the overall microbiota profiles from blood group A individuals, a shift towards higher %G + C microbes was observed, and the profiles from blood group B individuals showed the highest microbial density in the mid-%G + C area. In the high %G + C range, the highest peak was observed in the blood groups O and AB. The observed differences in the %G + C profiles were found to be statistically significant (Figure 2). The short chain fatty acid and lactic acid analysis or total bacterial numbers determined by flow cytometry did not differ between the ABO blood groups (data not shown).

**Table 2:** Statistical significances between 5%G + C-fractionated samples grouped and averaged by ABO blood group

**5% increment**	****A vs. O****	****A vs AB****	****A vs B****	****O vs B****	****O vs AB****	****AB vs B****
20-24	**0,0002**	**0,0020**	0,0605	**0,0012**	**0,0004**	**0,0021**
25-29	0,0396	**0,0001**	0,0194	**0,0004**	**0,0015**	0,1365
30-34	0,0064	**0,0003**	**0,00001**	**0,0002**	0,0356	0,2061
35-39	**0,0036**	**0,0036**	**0,00045**	0,0129	0,0576	0,1745
40-44	0,0887	0,0340	0,1827	0,0106	0,0150	**0,0003**
45-49	**0,0001**	**0,0004**	**0,0010**	**0,0019**	**0,0039**	**0,0001**
50-54	0,0370	0,0085	0,1061	**0,00001**	0,0058	**0,0004**
55-59	**0,0015**	0,0055	0,1444	0,0173	0,0171	0,0190
60-64	0,3104	0,0091	**0,0016**	**0,0016**	**0,0016**	**0,0051**
65-69	0,0112	0,0769	0,1741	0,2190	0,0772	0,0364
70-74	0,0096	0,2943	0,3287	0,0104	0,0068	0,3402

Statistical significances between indicated groups were tested using Student's t-test; highly significant values (*p* < 0.005) are marked in bold.

**Figure 3:**
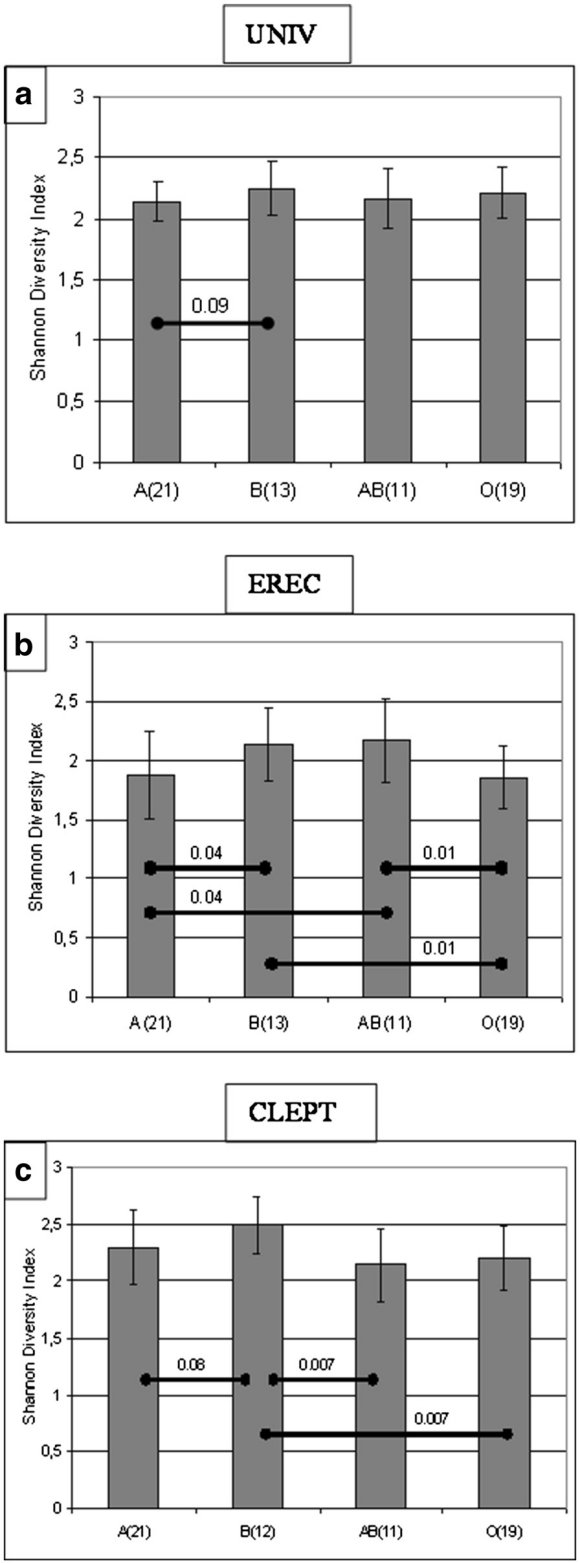
**ABO blood group related differences in the microbiota diversity.** The Shannon Diversity index calculations of the PCR-DGGE profiles obtained with a) universal eubacterial (UNIV) primers, b) *Eubacterium rectale* – *Clostridium coccoides* (EREC) primers and c) *Clostridium leptum* (CLEPT) primers. Columns are averaged ± SD values of the corresponding ABO blood groups. Statistically significant differences BASED on ANOVA tests between ABO blood groups are marked with diagonal bars and with the corresponding p-value.

A denaturing gradient gel electrophoresis (PCR-DGGE) analysis was performed to determine which major bacterial groups were responsible for the differences detected in the overall microbiota profile using %G + C profiling. The redundancy analysis (RDA) of the PCR-DGGE profiles revealed that ABO blood groups are statistically significantly associated with the intestinal microbiota composition, as determined by PCR-DGGE primers targeting all bacteria (UNIV: p = 0.015) and the *Eubacterium rectale – Clostridium coccoides* group (EREC: p = 0.032) (Figure2). The microbiota from subjects harbouring the B antigen (B and AB) differed significantly from non-B antigen blood groups (A and O) in regard to the levels of the UNIV (p = 0.005), the EREC (p = 0.005) and the *Clostridium leptum* (CLEPT) (p = 0.01) bacterial groups. In addition to the distinct clustering of the microbiota profiles, PCR-DGGE analysis revealed significant ABO blood group related differences in the species diversity within the EREC and the CLEPT groups, with blood groups B and AB showing the highest, and blood group O the lowest, diversity (Figure3). These findings suggest that the mucosal expression of blood group antigen B, in particular, appears to affect the dominant microbiota composition. The association of blood group B antigen is also reflected in the %G + C-range of 30–44.

The association we found between the ABO blood groups, especially the presence of the group B antigen, is strengthened by comparable results having been obtained using two broad-spectrum profiling methods. The semi-quantitative PCR-DGGE method identified specific associations within the major intestinal bacterial groups, and the qualitative %G + C profiling supported these findings and demonstrated that the microbial differences associated with the blood groups are large enough to affect the relative quantities of the major bacterial groups, thus impacting the overall microbial profile. We speculate that the statistically significant differences in these important bacterial groups may indeed have *in vivo* relevance. Besides adhesion sites, mucus provides endogenous substrates for bacteria in the intestine, especially in the colon, where the easily degradable carbohydrates have already been consumed [13,18,19]. Our present finding on the association of the blood group and the group B antigen with the composition of intestinal microbiota may partly help to explain the recent discovery of the three enterotypes of human intestinal microbiota [2]. Interestingly, an early study supports our result on the importance of the blood group B antigen: in 1976, Hoskins & Boulding published their findings showing that blood group B subjects had more B-antigen degrading glycosidases producing microbes in their faeces compared with other subjects [9].

**Figure 4:**
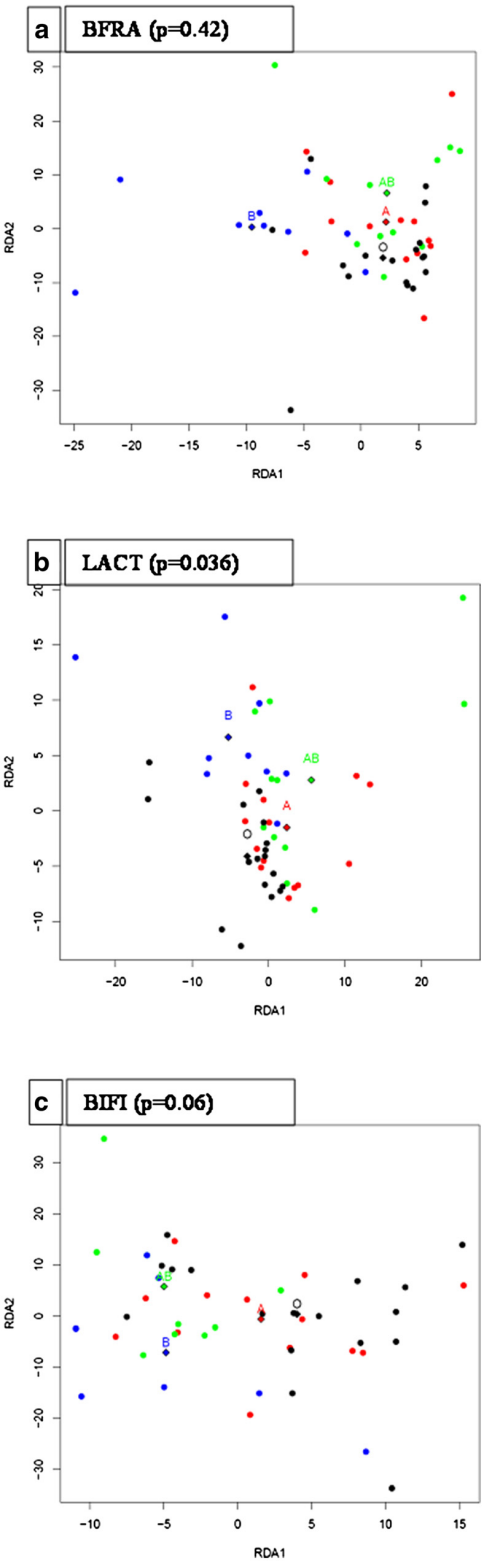
**RDA visualization of microbiota profile similarities and ABO blood group types.** Each dot represents a single individual, taking into account all individual intensities measured in each PCR-DGGE group. Diamonds mark the calculated data centre points of the corresponding blood groups. *P*-value marks the statistical significance of the differences between the blood groups from ANOVA-like permutation test. Dot colours for the ABO blood groups are as follows: A = red, B = blue, AB = green and O = black. a) PCR.-DGGE with *Bacteroides fragilis* (BFRA) primers, b) Lactobacillus (LACT) primers and c) *Bifidobacterium* (BIFI).

**Figure 5:**
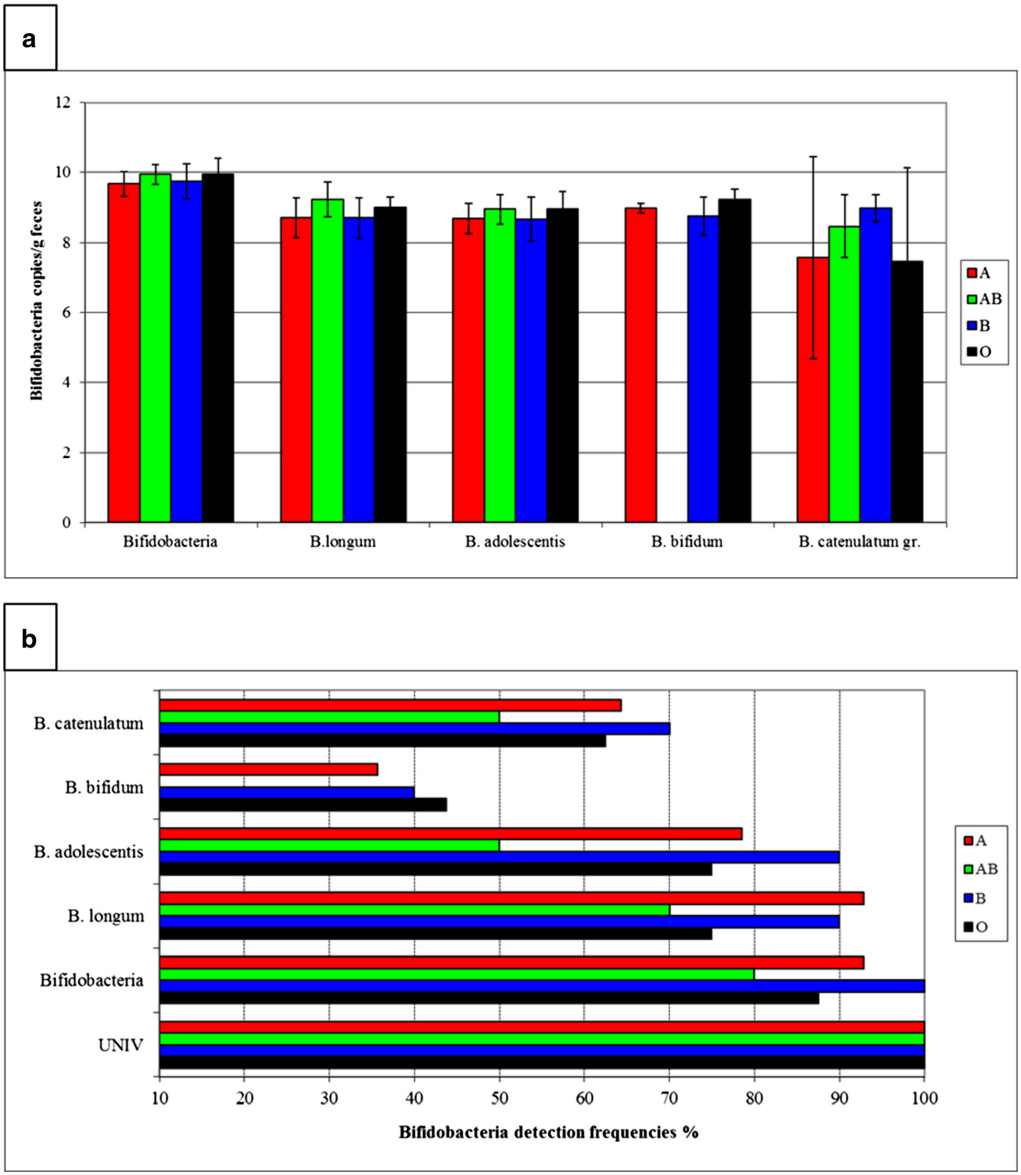
**Abundance of bifidobacteria in ABO blood groups. a)** Total bifidobacteria counts (copies/g faeces: average ± SD) by bifidobacteria species and genus specific qPCR-analysis. **b)** Detection frequencies (% of samples positive) of bifidobacteria as determined with the *Bifidobacterium* genus and species specific qPCR analysis.

**Figure 6:**
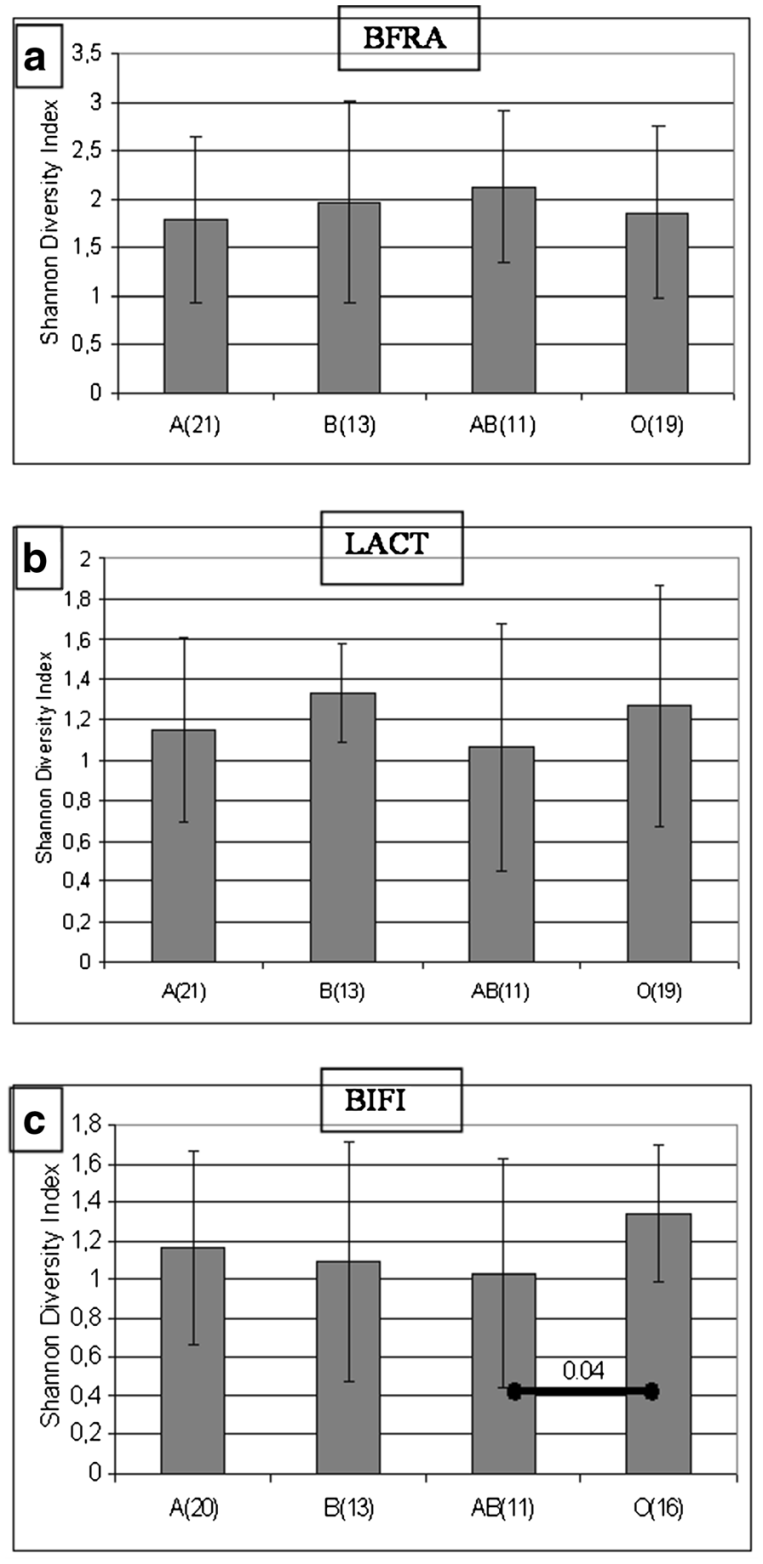
**ABO blood group related differences in the microbiota diversity.** The Shannon Diversity index calculations of the PCR-DGGE profiles obtained with **a)***Bacteroides fragilis* group (BFRA) primers, **b)***Lactobacillus* (LACT) primers and **c)***Bifidobacterium* (BIFI) primers. Columns are averaged ± SD values of the corresponding ABO blood groups.

To further explore the ABO blood group and ABO blood group antigen related associations in the intestinal microbiota, we continued microbiota profiling by targeting selected, less dominant bacterial groups colonising the intestine. Large individual variation in the diversity of the *Bacteroides* population was observed by BFRA DGGE. No ABO blood group related differences in the diversity or clustering of the *Bacteroides* population was observed (Figure4) even though *Bacteroides* spp. is known to be capable of utilising a variety of host-derived glycans, including blood group glycans [14]. We nevertheless observed certain ABO blood group associated differences in the detection frequency of some of the band positions in the BFRA DGGE (Figure 3), suggesting the existence of species or strain level differences in the *Bacteroides* population between the ABO blood groups. Since we observed ABO blood group related differences in the high GC area of the %G + C profile, we studied further the abundance of the genus *Bifidobacterium*, a typical component of the adult human intestinal microbiota, composing roughly 6% of the total microbiota [20]. The total average numbers of the genus *Bifidobacterium* in different ABO blood groups (Figure5) varied highly between the samples, and ABO blood group associated differences were not detected by the qPCR, when the results of blood groups were compared with ANOVA. In PCR-DGGE analysis blood group O subjects were observed to have higher diversity or clustering compared to blood group AB subjects (Figure6). As a culture-independent, yet primer-dependent, methods qPCR and PCR-DGGE rely on specificity and sensitivity of primers bacteria and %G + C-profiling is a solely culture-and primer-independent method allowing the detection of the most abundant microbial groups present in the sample regardless of prior knowledge of the groups, the differences between the bifidobacteria related results might be caused by both %G + C-detection of other *Actinobacteria* than *Bifidobacterium*, e.g. *Collinsella* species (second most abundant phylotype reported in *Actinobacteria*[21]), and qPCR/PCR-DGGE not detecting all possible bifidobacteria. Furthermore, the sudden disappearance of *B. bifidum* from AB-persons may be due to that *B. bifidum* is rather infrequently detected *Bifidobacterium* species in Caucasian adults [22] and thus the small number of study subjects may have influenced the result.

**Table 3:** Association of the bacterial PCR-DGGE genotypes with the ABO blood groups

	Detection frequency of the DGGE genotype**		
DGGE genotype*, number of genotypes	**B + AB vs. O + A (p-value)**	**A + AB vs. O + B (p-value)**	**O vs. A + AB + B (p-value)**
UNIV, 18.0%, 9	35% vs 3% (0.002)	6% vs. 22%	5% vs. 35%
UNIV, 31.4%, 21	48% vs. 23% (0.014)	38% vs. 28%	42% vs. 11%
UNIV, 32.2%, 8	30% vs. 3% (0.004)	13% vs. 13%	5% vs. 16%
UNIV, 33.8%, 56	74% vs. 95% (0.004)	84% vs. 91%	100% vs. 82%
UNIV, 39.0%, 9	17% vs. 13%	25% vs. 3% (0.026)	5% vs. 18%
UNIV, 42.2%, 9	30% vs. 5% (0.022)	16% vs. 13%	0% vs. 20%
UNIV, 47.0%, 7	22% vs. 5% (0.012)	9% vs. 13%	5% vs. 13%
UNIV, 49.4%, 8	0% vs. 20% (0.018)	13% vs. 13%	21% vs. 9%
UNIV, 58.8%, 11	30% vs. 8% (0.002)	16% vs. 19%	11% vs. 20%
UNIV, 61.1%, 17	17% vs. 0% (0.020)	9% vs. 3%	0% vs. 9%
LACT, 9.0%, 11	16% vs. 10% (0.092)	16% vs. 19%	11% vs. 20%
LACT, 14.1%, 15	26% vs. 18%	25% vs. 22%	5% vs. 31% (0.028)
LACT, 15.4%, 5	17% vs. 3 (0.072)	9% vs. 6%	0 vs. 11%
LACT, 66.3%, 10	17% vs. 15%	25% vs. 6% (0.082)	5% vs. 20%
LACT, 74.2%, 3	0% vs. 8%	0% vs. 9%	6% vs. 0% (0.023)
LACT, 83.1%, 4	9% vs. 0%	0% vs. 6%	0% vs. 4%
LACT, 84.7%, 40	65% vs. 59%	59% vs. 66%	74% vs. 58%
LACT, 86.6%, 3	0% vs. 8%	0% vs. 9%	16% vs. 0% (0.023)
LACT, 92.3%, 8	4% vs. 18%	6% vs. 19%	32% vs. 4% (0.007)
EREC 4.8%, n = 13	22% vs. 20%	34% vs. 6% (0.011)	5% vs. 27%
EREC 35.3%, 8	26% vs. 5% (0.048)	16% vs. 9%	5% vs. 16%
EREC, 39.7%, 9	26% vs. 5% (0.022)	16% vs. 13%	0% vs. 20% (0.048)
EREC, 46.9%, 19	52% vs. 18% (0.004)	31% vs. 28%	11% vs. 38% (0.004)
EREC, 50.9%, 34	70% vs. 43% (0.021)	53% vs. 53%	37% vs. 60%
EREC, 61.1%, 18	43% vs. 20% (0.044)	22% vs. 34%	32% vs. 27%
EREC, 73.9%, 28	61% vs. 35% (0.043)	44% vs. 44%	37% vs. 47%
CLEPT, 11.9%, 31	22% vs. 63% (0.002)	47% vs. 50%	63% vs. 42%
CLEPT, 15.4%, 8	22% vs. 8% (0.048)	6% vs. 19%	5% vs. 16%
CLEPT, 16.0%, 6	26% vs. 0% (0.002)	16% vs. 3%	0% vs. 13%
CLEPT, 20.5%, 9	26% vs.8% (0.022)	13% vs. 16%	5% vs. 18%
CLEPT, 38.8%, 8	22% vs. 8% (0.048)	16% vs. 8%	0% vs. 18%
CLEPT, 52.1%, 8	4% vs. 18%	9% vs. 16%	26% vs. 7% (0.044)
CLEPT, 67.9%, 12	30% vs. 13% (0.048)	13% vs. 25%	11% vs. 22%
CLEPT, 84.0%, 7	0% vs. 18% (0.037)	6% vs. 16%	26% vs. 4% (0.021)
BFRA, 5.0%, 5	21% vs. 0% (0.008)	6% vs. 9%	0% vs. 11%
BFRA, 9.9%, 10	21% vs. 13%	26% vs. 6% (0.043)	5% vs. 20%
BFRA, 21.5%, 9	25% vs. 10% (0.023)	6% vs. 22%	11% vs. 16%
BFRA, 36.8%, 7	0% vs. 18% (0.036)	10% vs. 13%	21% vs. 7%
BFRA, 62.8%, 5	0% vs. 13%	3% vs. 13%	21% vs. 2% (0.026)
BIFI, 26.6%, 40	59% vs. 77%	62% vs. 79%	94% vs. 61% (0.022)

*The DGGE analysis was performed by applying universal bacterial primers (UNIV) and specific primers for the lactic acid bacteria (LACT), *Eubacterium rectale – Clostridium coccoides* group (EREC), *Clostridium leptum* group (CLEPT), *Bacteroides fragilis* group (BFRA) and *Bifidobacterium* spp. (BIFI).

**Detection frequencies (% of samples positive) of the specified DGGE genotypes are presented. Statistical analysis: The Fisher's exact test based on band presence/absence data. P-values for the statistically significant differences are presented in parenthesis.

In our study, we did not observe any ABO blood group associated differences in the diversity or clustering of the *Lactobacillus* population (Figure6). However, the differences in the detection frequency of several *Lactobacillus* spp. genotypes (band positions: Figure 3) suggest the existence of specific ABO blood group associated *Lactobacillus* spp. species or strains as described by Uchida et al. [12].

The biochemical structures of the ABO blood group glycan antigens present in both platelets and secretory intestinal organs, including mucosal layer, were published already in 1952 [23]. Krusius et al. reported that ABO blood group antigens are present on erythrocyte glycoproteins as polyglycosyl chains [24]. Studies focusing on the expression of glycans in the human intestine have identified the presence of ABO type 1 glycans in the mucosal layer covering human orogastrointestinal tract and have shown that the fucosylated glycans, including ABO blood group glycan antigens, are detected less abundantly towards the distal parts of the intestine [16,17]. The ABO blood group glycans are reported to be exported to the mucus layer from goblet cells residing in the crypts of the small intestine [17]. *Secretor-* and *Lewis-*genes control the secretion of ABO blood group antigens to all bodily liquid secretions, such as tears, milk, saliva and gastrointestinal mucus, and to secreting organs, such as pancreas and liver (reviewed by Henry [25]). Already in 1960's and 1970's, correlations between human ABO blood group phenotype and susceptibility to develop several diseases were broadly postulated based on data from large epidemiological studies carried out around the world. Since the development of the high throughput genomic analysis tool, research has been increasingly focused on revealing correlations between individual genotypes and disease. Indeed, highly selective associations of ABO and Lewis blood group antigens as adhesion receptors have been described for common intestinal pathogen *Helicobacter pylori*[11], demonstrating the existence of genotype-specific bacterial adhesion on blood group glycan structures. However, the information on such interactions in commensal bacteria and their effects on the overall composition of the intestinal microbiota have been lacking.

## Conclusions

Here, we demonstrate that Finnish individuals with different ABO blood group status have differences in the repertoire and diversity of microbes of their intestinal bacterial population. In particular, the composition of the microbiota in individuals with B-antigen is differently clustered from that in non-B-individuals. We have also recently demonstrated differences in the intestinal microbiota composition associated with the host blood group secretor/non-secretor status [8]. These findings may at least partially explain the recent discoveries by Arumugam et al. [2] reporting clustering of human intestinal microbiota into three different enterotypes and by Wu et al. [3] correlating long-term diets to the enterotypes, as the host-microbe interaction in form of ABO blood group antigen dependent microbiota has not been taken into account in either of these studies. However, as all our study subjects were Caucasians from Finland, genetic variation being thus small between the subjects, the extrapolation of the results to international context would require additional samples from genetically and nutritionally differing areas. As our study provides a link between the host genetic factors and the clustering of the intestinal microbiota in this Finnish cohort, it also warrants further investigations with high-throughput techniques of microbiota analysis to evaluate whether the specific species/OTUs responsible for the microbiota differences can be found, thus potentially enabling new applications in the field of personalized nutrition and medicine.

## Methods

### Subjects and samples

One faecal and one blood sample was collected from 79 healthy Caucasian donors from Southern Finland for the analysis. Pregnant subjects and subjects with diagnosed GIT disorders, regular GIT complaints or antibiotic medication within two months prior to the faecal sampling were excluded from the study. All subjects were eating mixed diets and subjects on vegetarian diets were excluded. The nutritional intake was not controlled, except for not allowing drastic dietary changes or the habitual use of probiotic supplements/probiotic-supplemented food products and alcohol prior to the faecal sampling. Body mass index of the subjects was not calculated. The study was approved by the ethical committee of the Helsinki University Hospital and all subjects signed a written informed consent. Faecal samples were collected in containers with anaerobic atmosphere generators, samples were homogenized by mixing and distributed to 1 g aliquots in an anaerobic cabinet and aliquots were frozen at −70 °C within 5 hour from defecation.

The fecal aliquots were processed as described in [26] to isolate the bacterial genomic DNA. Briefly, 1 g of feces was washed to separate the eukaryotic cells from the microbial cells. The collected bacterial mass was pelleted with high speed centrifugation, the pellet was suspended to freeze-thaw buffer and the solution was frozen to −70 °C. A sample for flow cytometry was drawn at this stage. The sample for DNA extraction went through five freeze-thaw-cycles, after which enzymatic (lysozyme, proteinase K), chemical (sodium dodecyl sulphate) and bead beating techniques were utilized to break down the cells and chloroform-isoamylalcohol-extraction to isolate the bacterial genomic DNA from cell debris. The bacterial genomic DNA was purified using an isolation kit (Blood & Cell Culture DNA Midi Kit Cat no. 13343; Qiagen Inc., USA) according to manufacturer’s instructions. The isolated DNA was diluted to TE-buffer and the DNA concentration was determined using NanoDrop (Thermo-Fisher Scientific, USA). Quality of the DNA was assessed by measuring the ratio 260/280 nm, samples having ratio between 1.7-2.0 and total concentration higher than 20 μg/g were accepted.

Blood samples were analysed for the presence ABO and RH blood group with a haemagglutination assay using Olympus PK 7300 according to standard blood group typing practice. The secretor status of the individuals was determined based on the presence of Lewis a and Lewis b antigens by using monoclonal antisera (Sanquin, the Netherlands) and by genotyping of the *FUT2* gene as described in [8]. Volunteers with non-secretor phenotype (n = 15) were dismissed from further studies, resulting in a study group of 64 individuals (57 female and 7 male; age range 31–61 years). The demographic and blood group distribution of the volunteers is presented in Figure 1).

### Microbiota profiling by %G + C, SCFA and flow cytometry analysis

The genomic DNA in microbe samples was profiled using the %G + C-profiling technique allowing the identification of microbial clusters or subsets in samples according to their genomic G + C contents [26]. In brief, the method is based on the molecular weight difference between A-T and G-C linkages in DNA double helix, achieved by A-T binding dye bis-benzimidazole, enabling the separation of DNA strands with different AT/GC ratios by ultracentrifugation, which are then visualized using UV light. Samples with a low genomic DNA yield (<20 μg/g fecal material) were excluded from the analysis and the %G + C-profiling was performed for 46 samples (14 representing A, 16 O, 8 B and 8 AB blood group). The same subset of faecal samples was further analyzed using SCFA and flow cytometric analyses as follows. The analysis of SCFA and lactic acid was essentially performed as described by Fava et al. [27], using gas chromatography to establish the concentration of SCFAs acetic, propionic, butyric, isobutyric, valeric, isovaleric and 2-methylbutyric acids, as well as lactic acid. The total numbers of bacteria in the samples were determined using a flow cytometric FACSCalibur system (BD Biosciences, San Jose, CA, USA) as previously described in [28]. For the method, the samples were fixed with 37% formaldehyde to obtain final concentration of 4% and the samples were stained with a fluorescent nucleic acid binding dye, SYTO 24 (Molecular Probes, The Netherlands).

### PCR-DGGE analysis

An extended sample set consisting of faecal samples from 21 blood group A, 19 O, 13 B and 11 AB individuals was analyzed using PCR-DGGE targeting the dominant eubacteria (UNIV) and specific bacterial groups, namely *Eubacterium rectale* – *Clostridium coccoides* group (EREC); *Clostridium leptum* group (CLEPT); *Bacteroides fragilis* group (BFRA); *Bifidobacterium* spp. (BIF) and *Lactobacillus* spp. (LACT). The PCR-DGGE analysis was performed as described by [8], with bacterial group specific modifications. Briefly, DNA from 0.3 g of faecal material was extracted using the FASTDNA® SPIN KIT FOR SOIL (Qbiogene) and the quality of the DNA was determined using NanoDrop as described above. Partial eubacterial 16 S rRNA gene was amplified using PCR with universal or group-specific primers: UNIV, U-968-F-GC and U-1401-R [29], EREC, CcocF and CcocR-GC [30], CLEPT, Clept-F and CleptR3-GC [30], BFRA, BfraF and BfraR-GC [30], BIF, Bif164F and Bif662R-GC [30] and LACT, Lac1 and Lac2-GC [31]. Amplified PCR fragments were separated in 8% DGGE gel with denaturing gradient ranging from 45% to 60%. DGGE gels were run at 70 V for 960 min in a gradient optimised for each bacterial group (UNIV 38-60%, EREC 40-58%, CLEPT 30-53%, BFRA 30-45%, BIF 45-60% and LACT 38-55%). DGGE gels were stained with SYBRSafe for 30 mins and documented with SafeImager Bluelight table (Invitrogen) and AlphaImager HP (Kodak) imaging system. Digitalised DGGE gel images were imported to the Bionumerics-program version 5.0 (Applied Maths) for normalisation and band detection. The bands were normalised in relation to a marker sample specific for the said bacterial groups. Band search and band matching were performed as implemented in the Bionumerics. Bands and band matching were manually checked and corrected. The principal component analysis was calculated in the Bionumerics. The PCR-DGGE band intensity data was analyzed with Redundancy Analysis (RDA) [32] using ABO blood group status or presence of B-antigen as grouping factors followed by ANOVA-like permutation test.

### Bifidobacteria-specific qPCR

The qPCR method was applied to detect and quantify the 16 S rRNA gene copies of bacteria, bifidobacteria and four bifidobacterial species/groups, *B. bifidumB. longum* group, *B. catenulatum/pseudocatenulatum* and *B. adolescentis* in faecal samples [8]. In short, reaction mixture was composed of 0.3 μM of each primer, PCR Master Mix and faecal DNA diluted 1 ng/μl for bifidobacteria group/species-specific primer pairs and 0.1 ng/μl for universal primers and bifidobacteria primers. All the samples and standards were analyzed in three replicates. The results were compared to standard curves for each bacterial group of known concentrations of the bacterial genomic DNA (from 10 ng/μl to 0.0001 ng/μl) and calculated as copies/g wet feces and the detection threshold was set to 10^7^ copies/g. The amplification efficiencies were from 93% to 98% for all the other qPCR primer pairs except for *B. bifidum* specific primers, in which amplification efficiency varied from 80% to 92% and for *B. catenulatum/pseudocatenulatum*, in which efficiency varied from 87% to 91%.

## Competing interests

The authors declare that they have no competing interests.

## Authors’ contributions

HM and JM Designed and managed the study, organised the donor sample collection, analysed the data and wrote the article. SJL and MB designed and performed %G + C-profiling- and SCFA-analysis. PW performed PCR-DGGE-analysis and analysed the PCR-DGGE-data. ET performed PCR-DGGE-analysis. JN performed the bioinformatic analysis. HT supervised the blood group status measurements and analysed the results. ACO and KA were involved in study design. All authors read and approved the final manuscript.
